# The accelerometer-based navigation system demonstrated superior radiological outcomes in restoring mechanical alignment and component sagittal positioning in total knee arthroplasty

**DOI:** 10.1186/s12891-021-04213-9

**Published:** 2021-04-13

**Authors:** Jiaxiang Gao, Yunfei Hou, Rujun Li, Yan Ke, Zhichang Li, Jianhao Lin

**Affiliations:** 1 Arthritis Clinic & Research Center, Peking University People’s Hospital, Peking University, Beijing, 100044 China; 2 Arthritis Institute, Peking University, Beijing, China

**Keywords:** Total knee arthroplasty, Accelerometer-based navigation, Mechanical axis, Component sagittal positioning

## Abstract

**Background:**

This study aimed to determine whether the accelerometer-based navigation (ABN) could improve the accuracy of restoring mechanical axis (MA), component positioning, and clinical outcomes compared to conventional (CON) total knee arthroplasty (TKA).

**Methods:**

A total of 301 consecutive patients (ABN: 27, CON: 274) were included. A 1:4 propensity score matching (PSM) was performed between the two groups according to preoperative demographic and clinical parameters. The postoperative MA, femoral coronal angle (FCA), femoral sagittal angle (FSA), tibial coronal angle (TCA) and tibial sagittal angle (TSA) were compared. Absolute deviations of aforementioned angles were calculated as the absolute value of difference between the exact and ideal value and defined as norms if within 3°, otherwise regarded as outliers. Additional clinical parameters, including the Knee Society knee and function scores (KSKS and KSFS) and range of motion (ROM), were assessed at final follow-up (FU) (mean FU was 21.88 and 21.56 months respectively for ABN and CON group). A secondary subgroup analysis and comparison on clinical outcomes were conducted between norms and outliers in different radiological parameters.

**Results:**

A total of 98 patients/102 knees were analyzed after the PSM (ABN: 21 patients/24 knees, CON: 77 patients/78 knees). In the ABN group, the mean MA, FCA and TSA were significantly improved (*p* = 0.019, 0.006, < 0.001, respectively). Proportions of TKAs within a ± 3°deviation were significantly improved in all the postoperative radiological variables except for TCA (*p* = 0.003, 0.021, 0.042, 0.013, respectively for MA, FCA, FSA, and TSA). The absolute deviations of FSA and TSA were also significantly lower in the ABN group (*p* = 0.020, 0.048, respectively). No significant differences were found in either mean value, absolute deviation or outlier ratio of TCA between two groups. On clinical outcomes, there were no significant differences between two groups, although KSKS, KSFS and ROM (*p* < 0.01, respectively) dramatically improved compared to baseline. The subgroup analysis also demonstrated no statistical difference on clinical outcomes between the outliers and norms in varied radiological parameters.

**Conclusions:**

The ABN could improve the accuracy and precision of mechanical alignment and component positioning without significant improvement of clinical outcomes. Further high quality studies with long term FU are warranted to comprehensively evaluate the value of the ABN.

## Background

In total knee arthroplasty (TKA), both optimal mechanical alignment (MA) and component positioning will influence the outcomes [1]. It has been found that malalignment of greater than 3° would lead to apparent polyethylene wear [1] and premature failure [2], with a rising revision rate as high as 24% [3]. The accuracy of conventional technique on bone resection might be restricted in cases with obvious extra-articular deformities (EAD), excessive anterior femoral bowing and etc. [4]. The outliers ratio within these above mentioned challenging clinical scenarios may be as high as 22 to 35% [5].

With the aiming of improving accuracy and precision of overall limb alignment as well as component positioning in TKA, computer-assisted surgery (CAS) has rapidly developed and been well applied. Based on the Australian Orthopaedic Association National Joint Replacement Registry (AOANJRR), the rate of CAS navigation has increased from 2.4% in 2003 to 30.8% in 2016 [6]. Also De Steiger et.al found a significant decrease of the revision rate for aseptic loosening in the CAS-navigated group for patients < 65 years old over a 9-year follow-up (FU) [7]. Generally, three categories of CAS can be defined: image-based large-console navigation; imageless large-console navigation, and more recently, handheld accelerometer-based navigation (ABN) systems have been developed [8]. It has been demonstrated to provide a similar level of accuracy and precision at achieving a predefined alignment goal as large-console CAS, both of which are more accurate than conventional techniques using intramedullary or extramedullary instrumentation [9].

Disadvantages of large-console CAS includs higher economic costs, increased operative time, longer learning curve, and pin site complications (such as pain, wound drainage, infection, and rarely but devastatingly, fracture) [8]. While the typical workflow of handheld ABN devices provides a more similar feel to conventional intra/extramedullary alignment jigs and digital feedback as well as anatomical landmark referencing, with a minimal additional operation time and relatively low cost [10]. Previous studies [11–15] have evaluated the MA and component positions after TKA with ABN, the outlier rates of varied radiological parameters and clinical outcomes between navigation and conventional-technique groups have also been compared. However none of the previous studies evaluated the clinical outcomes between outliers and norms. The primary objective of this retrospective, propensity score-matched comparative study was to compare the postoperative radiological and clinical outcomes between patients undertaking TKA with the ABN system and conventional techniques. A subgroup analysis and comparison of clinical outcomes between outliers and norms have also been conducted as secondary objectives of the current study.

## Methods

### Patient selection

A retrospective review of institutional medical record database was conducted, which was approved by the local Ethics Committee of Peking University People’s Hospital (2020PHB171–01). Consecutive cases of patients who received a primary posterior-stabilized (PS) TKA by using either iAssist (Zimmer, Warsaw, IN) navigation system (ABN group) or conventional techniques (CON group) from May 2017 to September 2019 were included. Exclusion criteria included: (1) hip and/or ankle pathology with a severe limited range of motion (ROM) (2) or causing severe functional limitations, (3) patients lost to follow-up. The surgery was performed by one experienced arthroplasty surgeon (LJH).

### Data collection

Preoperative demographic data was collected. Preoperative Knee Society knee scores (KSKS) and function scores (KSFS) and ROM were also recorded.

### Surgical technique

The primary difference on surgical techniques exclusively existed in bone cutting, except for which, all other surgical procedures, i.e. approach (medial parapatellar approach), soft tissue balancing (gap balancing technique) and cementing techniques remained the same between both groups. In the ABN group, the surgeon followed the instructions based on the surgical technique manual of the iAssist system. A validation procedure was performed following every cut and make additional adjustments when necessary.

As for the CON group, standard intramedullary alignment technique on femoral side and extra-medullary alignment technique on tibial side were used. Preoperative radiographs, including weight-bearing anteroposterior (AP) view, lateral view and long-leg standing AP film, were referred to preoperatively.

### Radiological evaluation

Radiological assessment was carried out by utilizing standardized postoperative radiographs, including long-leg standing AP film, along with AP and lateral knee films [16]. All electronic radiographs were analyzed and measured by 2 independent observers (GJX and HYF) who had not participated in the surgery and been blinded to the allocation of groups. The radiographs were assessed twice, more than 2 weeks apart for each observer. The intra- and inter-observer reliability were evaluated and rated based on the method described by Konigsberg et al. [17]. The intra-observer reliability based on intra-class correlation coefficient (ICC) were 0.972 (95% CI: 0.965 to 0.978) and 0.942 (95% CI: 0.928 to 0.954), respectively for the two observers. For inter-observer reliability, the ICC was 0.953 (95% CI: 0.941 to 0.962).

**Fig. 1:**
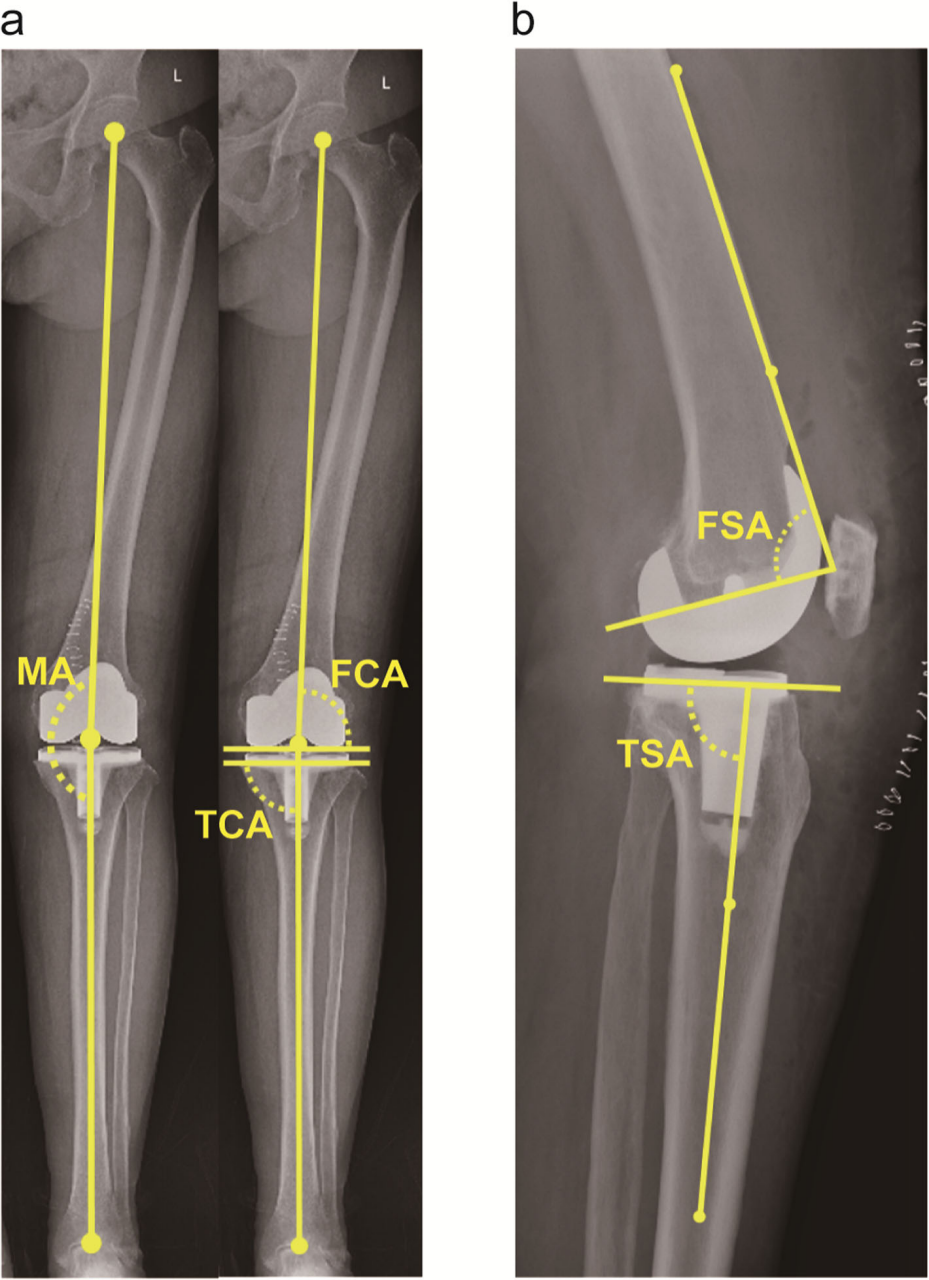
**a** Long-leg standing AP film for radiographic measurements in the coronal plane, including MA, FCA, and TCA. **b** Lateral radiograph for measuring component alignments in the sagittal plane, including FSA and TSA. Abbreviations, MA, mechanical axis; FCA, femoral coronal angle; TCA, tibial coronal angle; FSA, femoral sagittal angle; TSA, tibial sagittal angle

Three radiographic measurements were carried out on the AP hip-to-ankle radiographs (Fig. 1a): (1) lower extremity MA, which was formed by the angle bisecting the center of the femoral head, the center of the knee joint, and the center of the talus [12]; (2) femoral coronal angle (FCA), the lateral angle between femoral MA and intercondylar line; (3) tibial coronal angle (TCA), the medial angle between the tibial MA and the line parallel to the tibial tray. Two measurements were performed on the lateral films (Fig. 1b): (1) femoral sagittal angle (FSA), the posterior angle between the anterior cortical axis (the line linking two points of the anterior cortex at 5 and 15 cm proximal to the joint line [18]) of femur and the slope of distal femoral cut; (2) tibial sagittal angle (TSA), the posterior angle between the proximal anatomical axis (the line linking midpoints of outer cortical diameter at 5 and 15 cm distal to the knee joint [19]) of tibia and the slope of the proximal tibial cut. Absolute deviations of aforementioned angles were calculated as the absolute value of difference between the exact and ideal value (MA, 0°; FCA, 90°; TCA, 90°; FSA, 87°; TSA, 83° [20]). In line with the consensus of most research, absolute deviations of these angles were defined as norms if within 3°, otherwise they were regarded as outliers [21–23].

### Clinical evaluation

Clinical outcomes were assessed using the KSKS and KSFS [24], as well as ROM, which was measured by a goniometer on the basis of the active maximum knee ROM. All the clinical outcomes were collected by two co-authors (LZC and LRJ) who were blinded to the patient group.

### Propensity-matched analysis

Propensity score matching (PSM) analysis is a statistical technique aiming to minimize the effects of confounding factors attributable to measured covariates [25]. A propensity score was defined to be a patient’s conditional likelihood of being assigned a treatment based upon patient’s pre-treatment characteristics by logistic regression using the R (V.3.6.1) package “MatchIt” (V. 3.0.2) and “tableone” (V.0.10.0). In this particular study, PSM was conducted between the ABN group and the CON group with a 1:4 ratio, rather than a 1:1 ratio in order to increase precision [26]. A caliper of 0.02, based on age, gender, side of surgery, body mass index (BMI), ROM, KSKS and KSFS scores along with preoperative MA at baseline, was used in the PSM process.

### Statistical comparisons

Radiological and clinical data were compared between the ABN & CON groups. Clinical outcomes were also compared between outliers and norms in different radiological parameters, as secondary subgroup analysis. Continuous data (age, BMI, ROM, KSKS, KSFS, MA, FCA, TCA, FSA, and TSA), were presented as means ± standard deviations (SD) and compared by utilizing either Student’s t test (for normally distributed data with equal variances), Welch’s t test (for normally distributed data with unequal variances), or the Mann-Whitney U test (for non-normally distributed data). While the categorical data (gender, side of surgery, prosthesis type, and outlier ratio) were presented as counts and percentages, for which Fisher’s exact test (when expected count was less than 5) or Chi-Square-test (when expected count was no less than 5) was used to compare between groups. Specifically, gender, prosthesis type and outlier ratio of TCA were compared using Fisher’s exact test, while the rest of categorical variables were analyzed by Chi-Square-test. Pre- and post-operative comparisons were also conducted for clinical parameters using paired t-test. To adjust for the pre-operative baseline of clinical outcomes (KSKS, KSFS, and ROM), analysis of covariance (ANCOVA) was used to detect differences between groups. Statistical comparisons were conducted using SPSS (version 20.0; IBM SPSS Statistics, Chicago, IL, USA), with statistical significance set at *p* < 0.05.

## Results

### Patient characteristics

A total of 301 patients/332 knees were included in this study (ABN group: 27 patients/33 knees, CON group: 274 patients/299 knees). After PSM analysis, 98 patients/102 knees were finally included for analysis (ABN group: 21 patients/24 knees; CON group: 77 patients/78 knees). Demographic data before and after PSM were summarized in Table 1, revealing no significant differences in all demographic and clinical data after matching. By the time of final FU, there were no revision cases of any reasons within this group of 301 patients.

**Table 1:** Demographic characteristic of included patients

	Unmatched group		*P* value	Propensity-matched group		*P* value
	ABN group	CON group		ABN group	CON group	
Number of cases/ knees	27/33	274/299		21/24	77/78	
Age (years)	70.52 ± 6.50	69.36 ± 7.00	0.368	71.04 ± 6.75	69.18 ± 7.33	0.270
Gender (female)	31 (93.9%)	234 (78.3%)	0.033*	22 (91.7%)	68 (87.2%)	0.727
Knee (right)	16 (48.5%)	122 (40.8%)	0.507	10 (41.7%)	35 (44.9%)	0.967
Prosthesis type			0.040*			0.137
LPS-Flex	29 (87.9%)	198 (66.2%)		21 (87.5%)	51 (65.4%)	
Legion PS	2 (6.1%)	53 (17.7%)		1 (4.2%)	14 (17.9%)	
Attune PS	2 (6.1%)	48 (16.1%)		2 (8.3%)	13 (16.7%)	
BMI (kg/m2)	27.12 ± 2.87	26.88 ± 3.72	0.718	27.80 ± 2.85	27.40 ± 3.54	0.615
KSKS	42.24 ± 14.75	40.41 ± 16.17	0.534	40.79 ± 15.21	39.79 ± 16.26	0.790
KSFS	46.06 ± 22.94	52.93 ± 20.75	0.075	46.46 ± 19.31	49.88 ± 20.97	0.478
ROM (°)	95.15 ± 18.94	95.20 ± 19.90	0.989	95.83 ± 18.51	93.08 ± 22.15	0.582
MA (°)	4.38 ± 11.87	9.50 ± 11.87	0.021*	7.80 ± 8.76	8.04 ± 6.13	0.882
FU time (month)	21.79 ± 1.65	21.99 ± 8.03	0.754	21.88 ± 1.42	21.56 ± 8.66	0.725

Abbreviations: *BMI* Body mass index, *ROM* Range of motion, *KSKS* Knee society knee score, *KSFS* Knee society function score, *MA* Mechanical axis (neutral MA = 0°, valgus = negative, varus = positive), *FU* Follow-up

*P* value < 0.05 were strengthened by *

### Radiographic outcomes

Table 2 presented all the aforementioned radiological variables of interest. In the ABN group, proportions of TKAs within a ± 3° deviation were significantly higher in all postoperative radiological variables except for TCA (*p* = 0.003, 0.021, 0.042, 0.013, respectively for MA, FCA, FSA, and TSA). The absolute deviation of FSA and TSA were also significantly lower (*p* = 0.020, 0.048, respectively). The mean MA, FCA and TSA were significantly improved as well (*p* = 0.019, 0.006, < 0.001, respectively). In particular, no significant differences were found in all three statistical parameters of TCA between two groups.

**Table 2:** Radiographic outcomes between iAssist (ABN) group and conventional (CON) group

	ABN group (*n* = 24)	CON group (*n* = 78)	*P* value
MA value	0.32 ± 2.17	1.74 ± 3.38	0.019*
MA absolute deviation	1.85 ± 1.12	2.97 ± 2.36	0.058
MA within ±3° (n, %)	23 (95.8%)	51 (65.4%)	0.003*
FCA value	89.75 ± 2.22	91.66 ± 3.09	0.006*
FCA absolute deviation	1.66 ± 1.45	2.56 ± 2.38	0.145
FCA within 90 ± 3° (n, %)	22 (91.7%)	53 (67.9%)	0.021*
TCA value	89.53 ± 1.76	89.99 ± 1.82	0.287
TCA absolute deviation	1.38 ± 1.15	1.47 ± 1.07	0.507
TCA within 90 ± 3° (n, %)	22 (91.7%)	74 (94.9%)	0.624
FSA value	88.79 ± 1.90	87.69 ± 4.18	0.311
FSA absolute deviation	2.10 ± 1.49	3.37 ± 6.60	0.020*
FSA within 87 ± 3° (n, %)	18 (75.0%)	41 (52.6%)	0.042*
TSA value	83.22 ± 2.27	85.64 ± 3.50	< 0.001*
TSA absolute deviation	1.89 ± 1.21	3.39 ± 2.77	0.048*
TSA within 83 ± 3° (n, %)	21 (87.5%)	47 (60.3%)	0.013*

Abbreviations: *MA* Mechanical axis (optimal value = 0°, valgus = negative, varus = positive), *FCA* Femoral coronal angle (optimal value = 90°), *TCA* Tibial coronal angle (optimal value = 90°), *FSA* Femoral sagittal angle (optimal value = 87°), *TSA* Tibial sagittal angle (optimal value = 83°)

*P* value < 0.05 were strengthened by *

### Short-term clinical outcomes

All the clinical parameters significantly improved postoperatively comparing to the baseline in two groups (*p* < 0.001, p < 0.001, *p* = 0.002, respectively for KSKS, KSFS, and ROM in the ABN group; And *p* < 0.001 for all 3 parameters in the CON group). Nevertheless, no significant differences were found in mean postoperative KSKS, KSFS and ROM (Table 3) between two groups. As for the changes of these clinical parameters from baseline, the differences were also not statistically different between two groups (Table 3). In the subgroup analysis of short-term clinical outcomes between the outliers and norms in varied radiological parameters, including the MA and component positioning (FCA, TCA, FSA, TSA), no statistical difference were found (Table 4).

**Table 3:** Short-term clinical outcomes between iAssist (ABN) group and conventional (CON) group

	ABN group	CON group	*P* value
Clinical outcomes at final FU			
KSKS	90.38 ± 10.45	88.09 ± 13.81	0.487
KSFS	84.58 ± 14.14	84.48 ± 14.96	0.567
ROM	109.38 ± 13.70	109.71 ± 16.78	0.364
Changes of clinical outcomes from preoperative baseline			
KSKS	49.58 ± 15.34	48.30 ± 15.32	0.487
KSFS	38.13 ± 16.67	34.60 ± 16.48	0.567
ROM	11.79 ± 16.72	17.17 ± 16.94	0.364

Abbreviations: *KSKS* Knee society knee scores, *KSFS* Knee society function scores, *ROM* Range of motion

**Table 4:** Subgroup analysis on clinical outcomes between outliers and norms

	MA norms (within ±3°)	MA outliers	*P* value
KSKS	88.9 ± 12.7	88.0 ± 14.3	0.79
KSFS	84.7 ± 15.5	83.9 ± 12.5	0.53
ROM	109.1 ± 15.7	111.1 ± 17.2	0.45
	FCA norms (90 ± 3°)	FCA outliers	*P* value
KSKS	87.7 ± 14.3	91.3 ± 8.5	0.34
KSFS	83.9 ± 15.9	86.1 ± 10.8	0.81
ROM	109.0 ± 16.5	111.4 ± 14.7	0.57
	FSA norms (87 ± 3°)	FSA outliers	*P* value
KSKS	87.9 ± 13.9	89.6 ± 11.9	0.51
KSFS	82.8 ± 16.1	86.9 ± 12.3	0.24
ROM	109.2 ± 15.6	110.2 ± 16.8	0.82
	TCA norms (90 ± 3°)	TCA outliers	*P* value
KSKS	89.1 ± 12.8	83.3 ± 16.4	0.19
KSFS	84.7 ± 14.7	81.9 ± 15.1	0.56
ROM	110.0 ± 15.8	105.6 ± 19.0	0.55
	TSA norms (83 ± 3°)	TSA outliers	*P* value
KSKS	89.0 ± 11.6	87.9 ± 15.7	0.42
KSFS	84.0 ± 15.5	85.4 ± 13.2	0.80
ROM	109.0 ± 16.0	110.9 ± 16.4	0.58

Abbreviations: *KSKS* Knee society knee scores, *KSFS* Knee society function scores, *ROM* Range of motion, *MA* Mechanical axis, *FCA* Femoral coronal angle, *TCA* Tibial coronal angle, *FSA* Femoral sagittal angle, *TSA* Tibial sagittal angle

## Discussion

The current study demonstrated that with comparable clinical improvements in the ABN and the CON group, the iAssist system not only restored MA accurately and precisely, but also significantly improved prosthesis positioning, especially for the sagittal alignment of both femoral and tibial components.

With the exception of one study suggesting no significant difference in the ratio of outliers for lower limb alignment and component placement [14], the results of the present study were consistent with most published investigations comparing ABN with CON techniques [11–13, 15]. A prospective randomized controlled trial (RCT) found significant improvements in postoperative mean MA, FCA, and TCA, along with lower combined outlier ratios of femoral and tibial component (4.0% in the ABN group, in comparison with 32% in the CON group) [12], though the authors did not analyze the radiological variables in the sagittal plane. Nam et al. retrospectively compared the tibial component positioning between KneeAlign system, whose working rationale was similar to that of iAssist, with CON instrumentations, and observed fewer outliers for TCA and TSA as well [15]. Another retrospective comparative study also yielded similar results for restoring lower limb MA and achieving proper component positioning [13].

One crucial finding of the present was the superior efficacy of iAssist system for aligning the components in the sagittal plane. Sagittal plane positioning and alignment of component would affect patients’ functional outcomes and prosthesis survivorship. Compared with a neutrally aligned implant, a slight-to-mid flexed femoral component could reduce the patellofemoral (PF) joint contact stress in PS-TKA [27]. However, an over-flexed femoral component, especially in patients of short stature, was associated with increased occurrence of persistent flexion contracture [28] and also impingement may occur between the post- and the femoral component [27]. Conversely, extraordinary femoral component extension might cause anterior knee pain in the long term [29]. Kim et.al highlighted the effect of femoral component’s sagittal positioning on prosthesis survivorship and noted that a surgeon should intend to place the femoral component within 0–3° flexion in the sagittal plane, if not, outliers would impact the component survival at a mean FU of 15.8 years [30]. Navigation was helpful in achieving an appropriate femoral sagittal alignment. While the flexion of femoral component was highly varied in conventionally aligned TKA [31], and a recommended femoral sagittal alignment of within 3° flexion could be acquired in only 25% of the studied cases [32].

Studies have also demonstrated that the tibial slope related linearly to the postoperative ROM [33, 34], when the slope was within 10°, a 1° increase would lead to a 2.6° increase in the knee flexion angle for cruciate-retaining (CR) TKA [34]. While excessively increased tibial slope might cause a greater contact stress on the tibial post, leading to increased polyethylene wear [35] as well as anterior impingement between the post and the femoral component, observed at near-full extension [36]. Iorio et.al found that traditional instrumentations failed to achieve ideal tibial component positioning, with a tendency towards decreased tibial slope [37].

In the current study and similar investigations [12, 14, 38], there were outliers as well in the ABN group despite validation procedure being performed. Hasegawa et.al defined these deviations between the radiographic and intraoperative component angles as radiographic errors [39]. In their study, the mean absolute tibial radiographic error were 0.8 ± 1.0° and 1.3 ± 1.1° in the coronal and sagittal planes, respectively. While the mean absolute error of the MA was 1.9 ± 1.2°. There were several proposed reasons for these outliers. First, uneven cementation and impaction of components can introduce considerable errors regardless of how accurately the resection planes are made [40, 41]. Second, large movements of the thigh, especially adduction, during the femoral registration, will induce anterior lift of the femoral head [42], introducing hip center registration errors. Any movement of the pelvis during circumduction could cause hip center rotation definition errors too [43]. Additionally, a stiff hip due to any aetiology that precludes an unrestricted hip circumduction is another plausible cause of hip center errors [44], though in the current study, such patients were excluded for analysis. Similarly, errors in ankle center registration may be responsible for tibial component outliers [44]. Third, sometimes the surgeon had accepted residual malalignment during surgery as understood from the navigation data to obtain a balanced extension and/or flexion space. Fourth, the outliers could be created from unstable trackers. Lastly, for patients with tibia vara deformity, tibial component is likely to be in valgus alignment (approximately 1°) even if a neutral angle (0°) has been selected in the procedure [42]. The deformity could cause medial positioning of the tibial eminence against the tibial shaft, which would consequently produce a shift of the tibial MA identified by the system [45].

On clinical outcomes, we found no significant differences in KSS and ROM between two groups, which was consistent with existing literatures. Liow et.al found no differences in both KSS and Oxford Knee Score (OKS) between the iAssist and the conventional group at 6 months postoperatively [13]. Another cohort study also demonstrated no differences in ROM, KSS and OKS following TKA between the two groups at both 6 months and 2 year FU [38]. Additionally, we observed no statistical differences between outliers and norms in all radiographic parameters. These results may raise a concern that the outlier reduction might not lead to incremental improvements in clinical outcomes in short term, which would make scholars overlook the role of ABN or CAS. In this study, we have used the widely accepted standard to define outliers as deviations of 3°. However, 3° is an arbitrary figure rather than a genuine cut-off value for malalignment [21, 46]. A spectrum probably exists, with a higher probability of failure as malalignment increases, probably due to excessive strain, eccentric loading and subsequent polyethylene wear or trabecular collapse [47–49]. Considering the results of these laboratory studies, we cautiously hold positive attitudes towards the value of ABN TKA surgery, which might translate into improved survival in investigations with a long term FU. According to the AOANJRR report, a total of 114,859 navigated TKA with better postoperative component alignment showed a reduced 10-year revision rate and a significant reduction in revision secondary to component loosening [7]. Another study with 15.8-year FU also showed that malalignment was a risk factor for failure of the components in the long term [30].

The value of navigation could also lie on implementing individualized and kinematic alignment (KA) TKA, whose main purpose and rationale is to restore a patient-specific and native pre-arthritic knee alignment and anatomy [50–52]. Compared to the traditional MA technique, KA could achieve better PF [53] and tibiofemoral joint biomechanics [54] and thus better patient preference [55]. Furthermore, KA TKAs were able to achieve the same degree of sagittal correction as MA techniques with less bony resection and soft-tissue releases [56]. Additionally, the 10-year implant survival of KA TKA was not compromised [57].

**Fig. 2:**
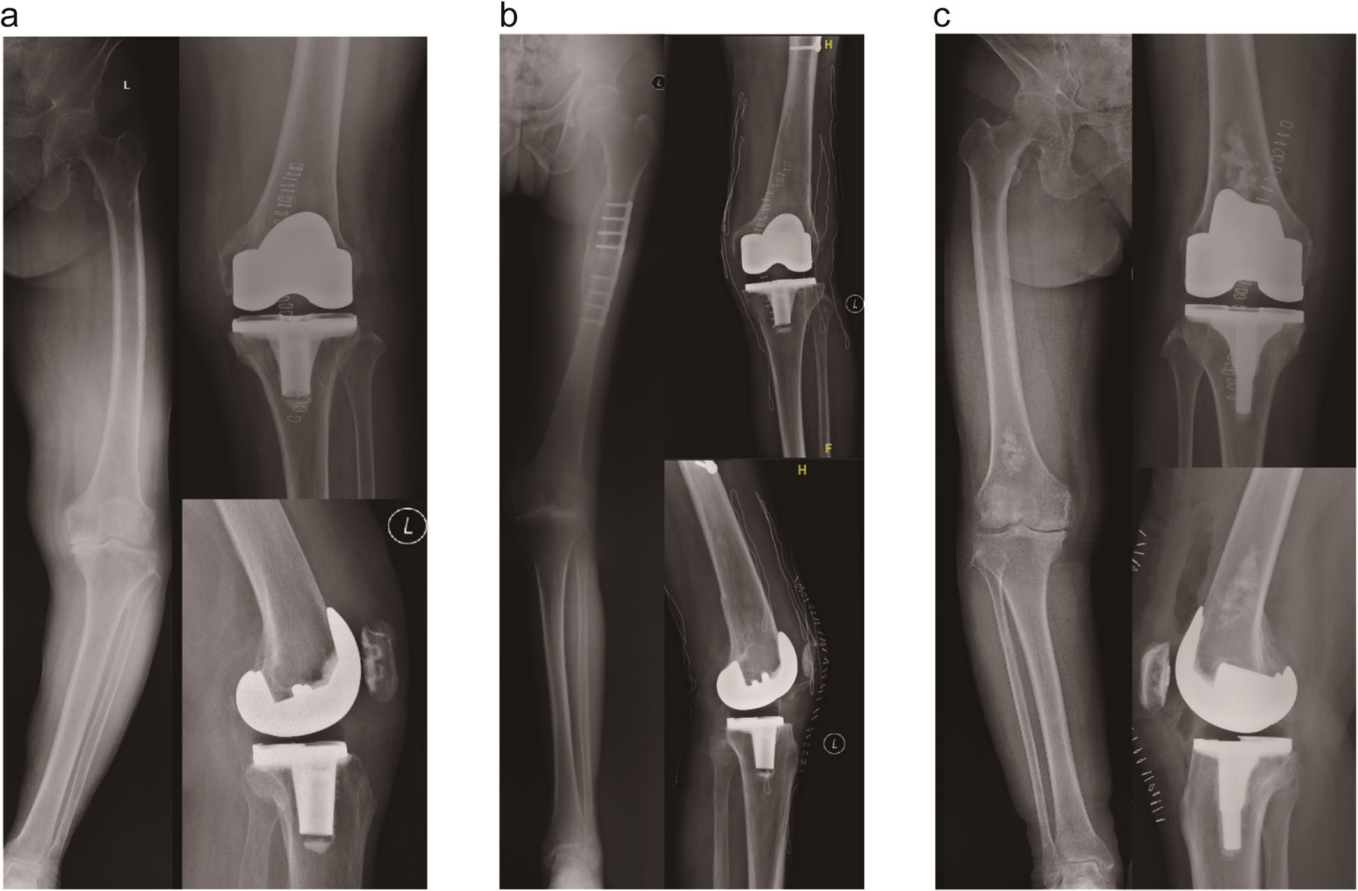
**a** Pre- and postoperative radiographs of patient with femoral EAD. **b** Pre- and postoperative radiographs of patient with lower limb fracture malunion and hardware retaining. **c** Pre- and postoperative radiographs of patient with benign bone tumor in distal femur

From the health economics standpoint, the value of new technology including computer navigation could be defined as the benefit divided by the cost [58]. Based on 2007 costings, Novak et al. employed a decision-analysis model to estimate the cost-effectiveness of CAS navigation in TKA, determining that cost-savings could be achieved in the long-term if the cost of CAS navigation was $629 or less per operation [59]. Thus selective use of the ABN would be an optimal strategy, we proposed several suitable indications of the ABN based on our clinical experiences and previous research. For patients with a femoral or tibial EAD, being accurate could be technically demanding [33], in which cases surgeons might fail to figure out the proper valgus cut angle of distal femur (Fig. 2a). In such cases, the ABN system appeared to be valuable [60]. Individuals with lower limb fracture malunion may develop EAD and/or have hardware (Fig. 2b). It was also a potential advantage of the ABN to obtain the desired component positions without irritating the medullary. In the similar rationale, the surgery was performed for one patient with benign bone tumor in distal femur uneventfully with the use of iAssist, without offending the tumor (Fig. 2c). Moreover, for individuals with extraordinary anterior femoral bowing, similar to those with an EAD, femoral component flexion would have a significant increase [61]. Utilising ABN could also make sense.

Another important strategy is to minimize the technical errors within navigation TKA, based on potential reasons for the errors, surgeons should make sure that trackers are always stable. Small movements without excessive forces during registration is recommended and lastly surgeons should perform a meticulous cementation technique. One should also notice that there are higher chances of creating technical errors in patients with a stiff hip or obvious tibia vara deformity.

There were several limitations of this study. First, it was a retrospective study in nature, and selection bias existed. We mitigate the negative effects by utilizing the PSM analysis, to minimize the inherent bias produced from covariates, and further lower the influence of confounding factors. Second, the FU period was relatively short. In order to explore the effect of ABN system in a more comprehensive fashion, future prospective studies focusing on radiological, functional and survival outcomes with longer FU are warranted. Third, only lateral knee films but not lateral long leg standing X-ray were available and used when assessing the FSA. Although the authors concluded that there were only small differences between anatomical and mechanical sagittal femur axes [14], using the short film would inevitably generate measurement errors. Finally, only two male patients undertook primary TKA with the iAssist system during May 2017 to September 2019, which seemed to be a coincidence accounting for this great gender disparity.

## Conclusions

Although clinical differences were not observed either between the ABN and CON group, or between the outliers and norms, the results of the current study demonstrated that the use of ABN aided in achieving better radiological results including postoperative MA and component positioning, especially for sagittal component alignment (FSA and TSA). Two essential strategies (selecting optimal patients and reducing technical errors) were provided and discussed in order to maximize the value of ABN in TKA.

## Data Availability

All of the data will be available for secondary analysis in necessary cases from the corresponding author through email address.

## Abbreviations

ABN: Accelerometer-based navigation
MA: Mechanical axis
CON: Conventional
TKA: Total knee arthroplasty
PSM: Propensity score matching
FCA: Femoral coronal angle
FSA: Femoral sagittal angle
TCA: Tibial coronal angle
TSA: Tibial sagittal angle
KSKS: Knee Society knee scores
KSFS: Knee Society function scores
ROM: Range of motion
FU: Follow-up
EAD: Extra-articular deformities
CAS: Computer-assisted surgery
AOANJRR: Australian Orthopaedic Association National Joint Replacement Registry
PS: Posterior-stabilized
AP: Anteroposterior
ICC: Intra-class correlation coefficient
SD: Standard deviations
ANCOVA: Analysis of covariance
RCT: Randomized controlled trial
PF: Patellofemoral
CR: Cruciate-retaining
OKS: Oxford Knee Score
